# Therapeutic potential of statins and the induction of heme oxygenase-1 in preeclampsia^☆^

**DOI:** 10.1016/j.jri.2013.12.120

**Published:** 2014-03

**Authors:** Wenda Ramma, Asif Ahmed

**Affiliations:** a Department of Pathology, Beth Israel Deaconess Medical Center, Harvard Medical School, Boston, MA 02215, USA; b Vascular Biology Laboratory, School of Medical Sciences, Aston University, Birmingham B4 7ET, England, United Kingdom

**Keywords:** Preeclampsia, Vascular endothelial growth factor (VEGF), Soluble Flt-1 (sFlt-1), Soluble Endoglin (sEng), Heme oxygenase-1 (Hmox1), Carbon monoxide (CO), Statins

## Abstract

Heme oxygenase (Hmox) is an endogenous system that offers protection against placental cytotoxic damage associated with preeclampsia. The Hmox1/carbon monoxide (CO) pathway inhibits soluble Flt-1 (sFlt-1) and soluble Endoglin (sEng). More importantly, statins induce Hmox1 and suppress the release of sFlt-1 and sEng; thus, statins and Hmox1 activators are potential novel therapeutic agents for treating preeclampsia. The contribution of the Hmox system to the pathogenesis of preeclampsia has been further indicated by the incidence of preeclampsia being reduced by a third in smokers, who had reduced levels of circulating sFlt-1. Interestingly, preeclamptic women exhale less CO compared with women with healthy pregnancies. Hmox1 is reduced prior to the increase in sFlt-1 as Hmox1 mRNA expression in the trophoblast is decreased in the first trimester in women who go on to develop preeclampsia. Induction of Hmox1 or exposure to CO or bilirubin has been shown to inhibit the release of sFlt-1 and sEng in animal models of preeclampsia. The functional benefit of statins and Hmox1 induction in women with preeclampsia is valid not only because they inhibit sFlt-1 release, but also because statins and Hmox1 are associated with anti-apoptotic, anti-inflammatory, and anti-oxidant properties. The StAmP trial is the first randomized control trial (RCT) evaluating the use of pravastatin to ameliorate severe preeclampsia. This proof-of-concept study will pave the way for future global RCT, the success of which will greatly contribute to achieving the United Nations Millennium Development Goals (MDG4 and MDG5) and offering an affordable and easily accessible therapy for preeclampsia.

## 1 Introduction

Preeclampsia is characterized by the *de novo* onset of hypertension and proteinuria after 20 weeks of gestation. If left untreated it can lead to debilitating and potentially eclamptic seizures that can cause coma and even death. According to the Preeclampsia Foundation, globally preeclampsia accounts for 76,000 maternal deaths and 500,000 infant deaths every year. Currently, the lack of effective pharmacological options to treat preeclampsia indicates that there is an unmet need for an affordable and easily accessible treatment to prevent maternal and infant mortality. Factors originating in the placenta are likely to be responsible for the condition, yet the exact etiology of the disorder is unknown. Currently, the only curative management strategy is the premature termination of pregnancy and the delivery of the placenta. Compelling data have pointed to maternal endothelial dysfunction as the central phenomenon responsible for the clinical signs of the disorder–hypertension and proteinuria (Roberts et al., 1989). Theories that have been proposed to cause endothelial dysfunction include poor placental vascular remodeling, oxidative stress (Roberts and Redman, 1993), excessive inflammation (Redman et al., 1999), and an imbalance in angiogenic factors (Ahmed, 1997). Among these, the imbalance in anti-angiogenic factors has emerged as the one phenomenon that is most strongly associated with the clinical signs of preeclampsia and disease severity (Ahmed, 1997; Maynard et al., 2003; Venkatesha et al., 2006; Levine et al., 2005). In contrast, the elevation in inflammatory status observed in preeclampsia does not precede the onset of the disorder (Djurovic et al., 2002; Kronborg et al., 2011). In addition, an increase in inflammation is not associated with the increase in anti-angiogenic factors (Ramma et al., 2012) or disease severity (Ozler et al., 2012). Together, these do indeed indicate that the increase in inflammation occurs as a consequence of preeclampsia and that it is not the cause of the disorder (Ramma and Ahmed, 2011). To date, it seems that therapeutic strategies aimed at addressing the angiogenic imbalance in preeclampsia will provide the most promising outcome.

## 2 Angiogenic growth factors in the placenta

The human placenta is a rich source of angiogenic growth factors. Indeed, numerous studies have demonstrated the expression and localization of various vascular growth factors and their receptors in the placenta (Sharkey et al., 1993; Charnock-Jones et al., 1994; Ahmed et al., 1995; Kilby et al., 1996; Khaliq et al., 1996, 1999; Dunk and Ahmed, 2001). However, the finding that vascular endothelial growth factor (VEGF) stimulates nitric oxide release from trophoblasts and endothelial cells *via* the VEGF receptor-1 (Flt-1) (Ahmed, 1997) has led to a new approach to tackling preeclampsia.

Vascular endothelial growth factor (VEGF) is known to maintain endothelial cell integrity (Deanfield et al., 2007). The antagonist of VEGF, soluble Flt-1, binds to free VEGF and placental growth factor (PlGF) sequestering them and disrupting their proper signaling. The hypothesis that preeclampsia might arise because of the loss of VEGF activity as a result of the “increase in the levels of endogenous soluble Flt-1 that may antagonize the beneficial effects of VEGF” was first proposed in a review in 1997 (Ahmed, 1997). In recent years, clinical studies have shown that several weeks prior to the onset of preeclampsia, the levels of the anti-angiogenic factors, sFlt-1 (Levine et al., 2004) and sEng (Venkatesha et al., 2006) are elevated in the maternal circulation. Additionally, the maternal PlGF level is reduced many weeks before the onset of the disorder (Levine et al., 2005). Restoration of the angiogenic balance is critical to improving and even potentially curing preeclampsia. As proof, the *in vitro* removal of sFlt-1 from the placental condition media of preeclamptic women by immunoprecipitation restored the angiogenic imbalance and promoted the capillary-like tube formation similar to normal pregnancy (Ahmad and Ahmed, 2004). Furthermore, using the murine model of sFlt-1-induced preeclampsia, neutralization of sFlt-1 below a critical threshold eliminated the signs of preeclampsia in the mice (Bergmann et al., 2010). Clinically, the same concept of removal of sFlt-1 to improve preeclampsia was elegantly tested in severely preeclamptic women in whom the extractions of proteins by plasma apheresis resulted in a one-third reduction in circulating sFlt-1, accompanied by a decrease in proteinuria and stabilization of maternal blood pressure, leading to an increase in gestational age (Thadhani et al., 2011). Collectively, these separate studies do indeed demonstrate that restoration of the angiogenic balance correlates with the improvement in the clinical signs of preeclampsia.

## 3 Heme oxygenases

**Fig. 1:**
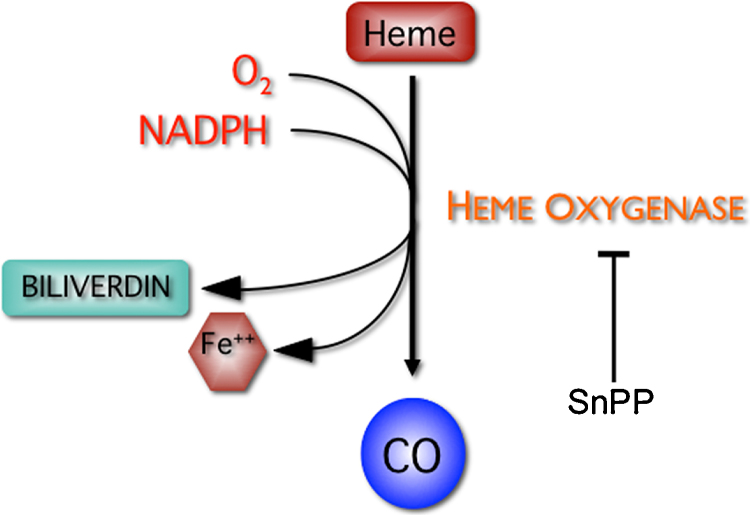
A schematic diagram of the heme oxygenase pathway. Heme oxygenase degrades heme to produce biliverdin, carbon monoxide (CO), and free iron. Biliverdin is rapidly converted to bilirubin by biliverdin reductase (BVR).

Heme oxygenase (Hmox) is the rate-limiting enzyme responsible for the degradation of heme in the endoplasmic reticulum to generate an equimolar amount of biliverdin, free iron, and carbon monoxide (CO) (Tenhunen et al., 1969). The pathway also consists of the co-factors, reduced nicotinamide dinucleotide phosphate (NADPH), molecular oxygen, and NADPH cytochrome P450 reductase, which, together with heme oxygenase, induce the catalysis of heme (Maines, 1988) (Fig. 1). Biliverdin is rapidly reduced to bilirubin, a potent anti-oxidant, by the cytosolic enzyme biliverdin reductase. CO is a potent vasodilator and also has anti-apoptotic properties. Hmox exists in two main isoforms, Hmox1 and Hmox2. The enzyme Hmox2 is a 36-kDa protein that is constitutively expressed at high concentrations in the brain, testis, and vascular endothelium. The inducible form, Hmox1 is a 32-kDa protein that is widely distributed in the body, with a high concentration in the liver and the spleen.

The major site of heme degradation in adult animals is the spleen, where a markedly elevated activity of Hmox is observed. In mammalian tissues, Hmox1 is induced by its substrate heme and also by heavy metals. It is also known as an oxygen-regulated protein-33 (Murphy et al., 1991) suggesting that oxygen concentration in tissues may influence its expression. Indeed, stimuli that cause oxidative stress, such as peroxynitrite, modified lipids, hypoxia, hyperoxia, ischemia/reperfusion, hyperthermia, and endotoxic shock, upregulate the expression of Hmox1 (Sikorski et al., 2004). There is mounting evidence for the beneficial functions of Hmox *via* its products (biliverdin, bilirubin, carbon monoxide, free iron), which regulate important biological processes including oxidative stress, inflammation, apoptosis, and angiogenesis in various conditions (Dulak et al., 2008). Deficiency of Hmox1 in humans results in severe and persistent endothelial damage, as indicated by the marked elevation in thrombomodulin and von Willebrand factor (Yachie et al., 1999). Decreased expression of Hmox1 has also been associated with pregnancy disorders, such as recurrent miscarriages (Zenclussen et al., 2006), intrauterine growth retardation (Zhao et al., 2009), and preeclampsia (Ahmed et al., 2000). This review will focus on the role of Hmox system in pregnancy, specifically in preeclampsia, where its beneficial role in improving the clinical signs of preeclampsia in animal models of preeclampsia has now been demonstrated by a number of independent laboratories.

### 3.1 Localization of Hmox in the placenta

Factors that trigger the clinical signs of preeclampsia originate from the placenta. Confirmation of the presence of Hmox in the placenta is important to determine its role in preeclampsia. Indeed, over the last two decades, the expression and localization of Hmox1 proteins within the placenta have been extensively investigated. In the rat placenta (Ihara et al., 1998) and the human placenta (Ahmed et al., 2000; McLean et al., 2000; Lash et al., 2003; Yoshiki et al., 2000), Hmox1 and Hmox2 are expressed at both the mRNA level and the protein level. Immunohistochemical analysis localized the expression of Hmox to the syncytiotrophoblast and the vascular endothelium in the placenta of uncomplicated pregnancy (Ahmed et al., 2000; McLean et al., 2000; Lash et al., 2003). Hmox2 is located in the endothelial cells and smooth muscle cells of the blood vessels of the placental villi (Yoshiki et al., 2000; Ahmed et al., 2000). During development, the expression of Hmox1 mRNA in the placenta was shown to gradually increase in parallel with gestational age (Ahmed et al., 2000; Miyagami et al., 2013). In contrast, Yoshiki and colleagues demonstrated that the mRNA and protein expressions of Hmox1 in human chorionic villi in the first trimester of pregnancy were similar to those of term in the placenta of uncomplicated pregnancy, whereas the expression of Hmox2 is increased in late pregnancy (Yoshiki et al., 2000). Despite reporting distributional differences, these studies demonstrate that the Hmox system is present within the placenta and is likely to play a functional role during pregnancy. Indeed, a recent study by Zenclussen's group showed that CO may act as a key molecule in pregnancy success by modulating uterine natural killer cells, which results in promotion the remodeling maternal spiral arteries (Linzke N, Schumacher A, Woidacki K, Croy BA, Zenclussen AC 2013. Carbon Monoxide Promotes Proliferation of Uterine Natural Killer Cells and Remodeling of Spiral Arteries in Pregnant Hypertensive Heme Oxygenase-1 Mutant Mice. December 23, 2013, doi: 10.1161/HYPERTENSIONAHA.113.02403).

### 3.2 Functional role of Hmox in the placenta

Through the production of its protective products (carbon monoxide, iron, and biliverdin), the Hmox system is able to provide mechanisms for protection during pregnancy against a variety of physiological threats. Hemoproteins are responsible for the transfer of oxygen from the mother to the fetus during pregnancy and heme is produced by the trophoblast, the site of exchange of substances between maternal blood and fetal circulation. The placental vascular endothelium and syncytiotrophoblasts are exposed to high concentrations of hemoglobin owing to their direct contact with fetal and maternal blood. As has been previously demonstrated, hemoglobin and free heme can undergo auto-oxidation to produce superoxide and hydrogen peroxide, which in turn promote the formation of reactive oxygen species (ROS) and damaging free radicals (Motterlini et al., 1995). Because of its direct contact with fetal blood, fetal heme is likely to be degraded in the syncytiotrophoblast by the Hmox system to release bilirubin and CO in the maternal circulation, hence contributing to fetal vasodilation of the placental villi (Ihara et al., 1998).

The syncytiotrophoblast is also directly exposed to maternal blood and to maternal immunological attack and other inflammatory stressors such as tumor necrosis factor-α (TNF-α), interferon-γ, and interleukins. These inflammatory mediators and other stressors may induce the expression of Hmox1 in the syncytiotrophoblasts to counteract the injurious effect of inflammation on the placenta and to prevent oxidative injury. Indeed, the pharmacological induction of Hmox1 in human placental villous explants was shown to offer placental cytoprotection (Ahmed et al., 2000). In preeclampsia, this endogenous protective system could be impaired and it is possible that the decreased expression or the loss of Hmox activity might contribute to the maternal endothelial dysfunction observed.

### 3.3 Hmox1 negatively regulates anti-angiogenic factors

In recent years, the functional benefit of Hmox in preeclampsia has gained increasing importance, in particular, after the publication demonstrating that the adenoviral overexpression of Hmox-1 or direct exposure to CO reduce both basal and VEGF-E-stimulated sFlt-1 release from human umbilical vein endothelial cells (HUVEC), while siRNA-mediated Hmox1 knockdown increases sFlt-1 release (Cudmore et al., 2007). Another study showed that the Hmox1 mRNA was decreased in the chorionic villous (fetal placental cells) sampling at just 11 weeks’ gestation in women who went on to develop preeclampsia compared with normal pregnancies (Farina et al., 2008). Furthermore, a recent study demonstrated that in samples of villous trophoblasts obtained from women between 6 and 11 weeks’ gestation of elective abortion, the mRNA levels of Hmox1 was significantly increased with gestational age, whereas the mRNA expression of sFlt-1 was significantly decreased with increasing gestational age (Miyagami et al., 2013). Collectively, these two studies reinforce the theory that during pregnancy, Hmox1 negatively regulates sFlt-1 and that the loss of Hmox1 activity early in pregnancy could be partly responsible for the cascade of events observed subsequently in preeclamptic pregnancies, such as the elevation in sFlt-1 as well as the increase in oxidative stress or inflammation (Ahmed, 2011).

Other laboratories have used mice models to demonstrate the effect of the loss of function of the Hmox1 system on pregnancy. Crossbreds of Hmox1 heterozygote mice (Hmox1^+/−^) resulted in Hmox1^+/−^ placentas that had significantly lower Hmox1 mRNA and protein levels compared with wild-type littermates. Additionally, the Hmox1^+/−^ placentas were smaller than the wild-type placentas. More importantly, the diastolic blood pressure and plasma sFlt-1 level were significantly elevated in pregnant Hmox1^+/−^ mice compared with their pregnant wild-type counterparts (Zhao et al., 2009). A recent study demonstrated that the pharmacological inhibition of Hmox-1 by tin mesoporphyrin in pregnant rats caused an increase in the maternal mean arterial blood pressure and a reduction in placental VEGF (George et al., 2013). These studies support the hypothesis that a partial deficiency in Hmox1 during pregnancy is associated with changes in the morphology of the placenta and the dysregulation in the angiogenic balance (Zhao et al., 2009; Zhao et al., 2011).

The reduced uterine perfusion pressure (RUPP) hypertension model, in which chronic RUPP leads to endothelial dysfunction and hypertension in the pregnant rat, has also been used as a tool to study the preeclamptic syndrome. Gilbert and colleagues demonstrated that the expression of Hmox1 in the placenta of the RUPP pregnant rats during placental ischemia was significantly reduced compared with control non-ischemic rat placentas. Interestingly, elevation in both circulating sEng and placental expression of sEng was observed in these pregnant rats (Gilbert et al., 2009). To substantiate the beneficial and therapeutic role of Hmox1 and its metabolites in preeclampsia, the same group demonstrated that the pharmacological induction of Hmox1 in this rat model attenuated the elevation in blood pressure, restored the angiogenic balance, and also reduced placental oxidative stress in the ischemic placenta (George et al., 2011). Furthermore, in the same RUPP model, George and colleague demonstrated that RUPP increased oxidative stress and promoted injury of the placenta by increasing the phosphorylation of mediators of injury in the placentas of pregnant rats. However, upon induction of Hmox-1 by cobalt protoporphyrin, an augmentation in pro-survival mediators (Erk and Stat3) was observed, resulting in injury attenuation (George and Arany, 2012).

**Fig. 2:**
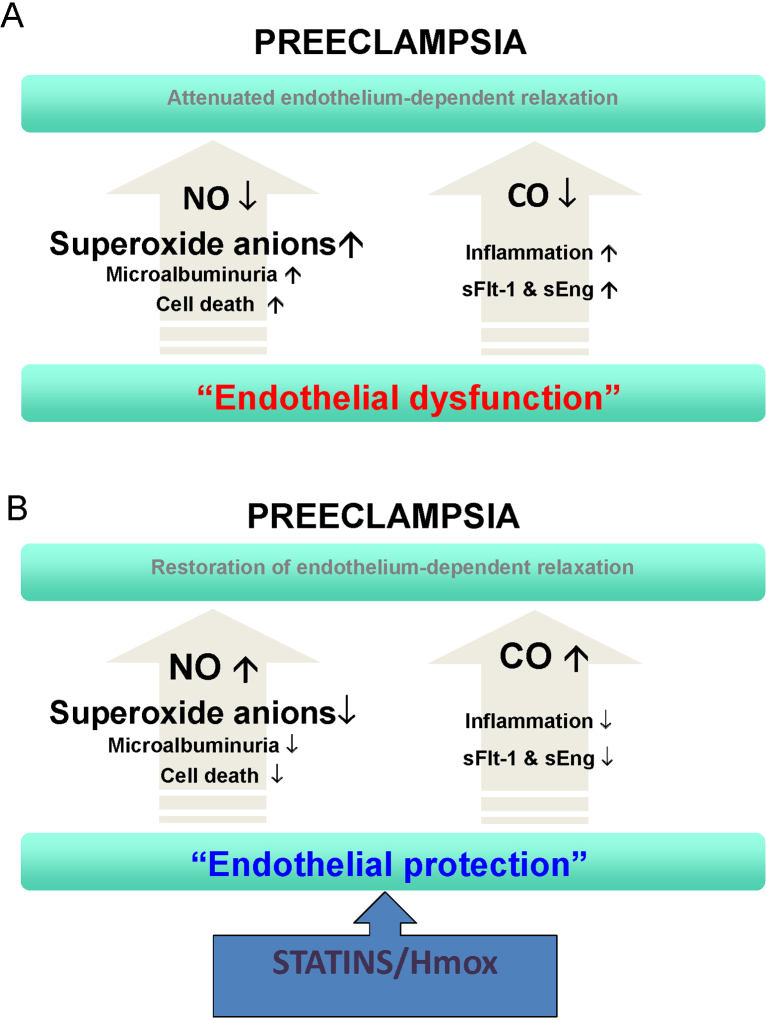
Unifying preeclampsia model of endothelial dysfunction and endothelial protection in the amelioration of preeclampsia. It is the loss of heme oxygenase-1 (Hmox1)/carbon monoxide (CO) activity, which leads to an increase in soluble Flt-1 (sFlt-1) and soluble Endoglin (sEng), that causes loss of nitric oxide (NO) bioavailability, giving rise to endothelial dysfunction and the preeclamptic phenotype. Decrease in CO and a rise in sFlt-1 may also exacerbate inflammation. Induction of Hmox1 or statins offers therapeutic benefit to patients by inhibiting sFlt-1 and sEng and stimulating Akt, NO, and CO.

In addition, a study by Zhou and colleagues demonstrated that angiotensin receptor-1 autoantibody (AT_1_-AA) from preeclamptic women injected into pregnant mice mediated the release of TNF-α, which subsequently induced the release of circulating sEng and sFlt-1 to impair placental angiogenesis. Of importance, this study also confirms that the pharmacological induction of Hmox1 by hemin prevented TNF-α from inducing sEng production in mice and that it also blocked the ability of serum obtained from pregnant mice injected with preeclamptic IgG to stimulate sEng production by human endothelial cells (Zhou et al., 2010). This indicates that the function of Hmox1 is downstream of AT_1_-AA-mediated TNF-α signaling to negatively regulate sEng production. The similar protective effect of PPAR-gamma in the RUPP model was mediated *via* the Hmox pathway (McCarthy et al., 2011). Based on these studies, it is fair to conclude that the ability of the Hmox pathway to beneficially affect multiple pathological pathways and restore vascular function during pregnancy makes it an attractive therapeutic target for treating preeclampsia (Fig. 2A and B).

### 3.4 CO and bilirubin in preeclampsia

One of the metabolites of the Hmox system, CO, has also been implicated in preeclampsia. The source of CO produced in the body, including in the human placental chorionic villi, was shown to originate from heme, primarily through the action of Hmox (Ahmed et al., 2005). Interestingly, women with preeclampsia have a significantly reduced level of CO in their exhaled breath compared with those with healthy pregnancies, indicating decreased Hmox activity (Baum et al., 2000; Kreiser et al., 2004). Furthermore, smoking during pregnancy reduces the incidence of preeclampsia, despite being associated with spontaneous abortion, stillbirth, preterm labor, fetal growth restriction, and placental abruption (Conde-Agudelo et al., 1999). Smokers are also known to have a reduced level of circulating sFlt-1 and increased placental growth factor (PlGF) (Levine et al., 2006). This paradoxical effect of smoking on the angiogenic factors could be explained by the experimental observation that exposure to CO reduces endothelial and placental sFlt-1 and sEng release (Cudmore et al., 2007). Furthermore, cigarette smoke extract has been shown to induce the expression of Hmox1 in placental explants (Sidle et al., 2007) and decrease sFlt-1 release from placental villous explants without affecting the placental apoptotic status (Mehendale et al., 2007). Taken together, these studies indicate that CO from cigarette smoke could account for the reduced incidence of preeclampsia in smokers through the inhibition in the release of sFlt-1 and increase in PlGF (Farina et al., 2008; Ahmed, 2011; Zhao et al., 2009).

To further emphasize the beneficial role of Hmox and its metabolites in preeclampsia, a recent retrospective observational study of 50,712 pregnancies, 925 of which involved preeclampsia, demonstrated that low levels of bilirubin were associated with poor maternal and infant outcomes in women diagnosed with preeclampsia, indicating that bilirubin may act as an anti-oxidant in this condition and thus modify the disease (Breslin et al., 2013).

### 3.5 Therapeutic potential of Hmox

A large amount of evidence has now confirmed that the reduction of sFlt-1 below a critical threshold is of benefit to preeclamptic women and those protective factors from the Hmox pathway could be the way forward in finding a therapy against preeclampsia. Indeed, agents capable of compensating for the deficiency or inducing the activity of the Hmox system to reduce sFlt-1 and sEng may have a good therapeutic potential in the treatment of preeclampsia. Based on this concept, Ahmed and co-workers have initiated a proof-of-concept clinical trial with pravastatin called the StAmP study in the United Kingdom (EudraCT number 2009-012968-13, clinicaltrialsregister.eu) (Ahmed and Williams, 2009). The main objective of the trial is to determine if pravastatin will lead to a significant reduction in circulating anti-angiogenic factors in women with early-onset preeclampsia. This trial will also reveal the beneficial or adverse clinical effects to the mother or the baby following gestational exposure to pravastatin.

**Fig. 3:**
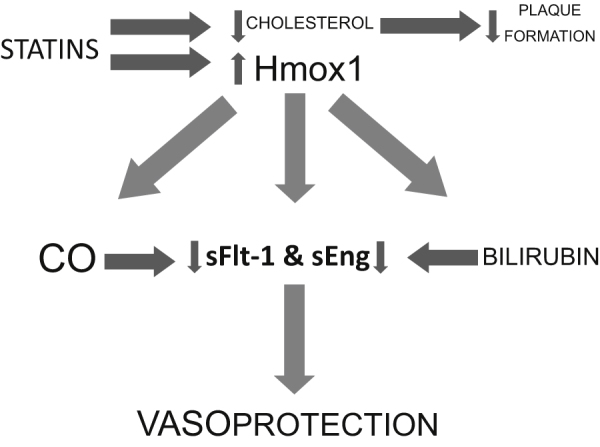
Schematic diagram of the benefits of heme oxygenase-1 and statins. Statin administration not only decreases cholesterol levels, leading to a decrease in plaque formation, but also induces heme oxygenase-1 (Hmox1) expression, which in turn leads to the subsequent production of carbon monoxide (CO) and bilirubin, which inhibit soluble Flt-1 (sFlt-1) and soluble Endoglin (sEng). This may be one of the mechanisms by which statins exert their vasoprotective actions.

Statins are primarily used to treat hypercholesterolemia by inhibiting the enzyme HMG-CoA reductase in the liver, thereby lowering LDL cholesterol production (Goldstein and Brown, 2009). They also exert a protective effect on vascular endothelial cells (Ludman et al., 2009; Weis et al., 2002). More importantly, statins induce the expression of Hmox1 and inhibit the cytokine-mediated release of sFlt-1 in cultured placental explants (Cudmore et al., 2007). Mice treated with statins were shown to have an increase in Hmox activity measured as an elevation in CO release from their tissues. Increased levels of plasma antioxidant were also observed in the same mice treated. Interestingly, treatment with an Hmox inhibitor, in the presence of statins, abrogated the increase in antioxidant levels in the mice, indicating that the Hmox pathway may mediate the beneficial pleiotropic actions of statins (Muchova et al., 2007) (Fig. 3).

Statins are currently contraindicated during pregnancy, although available information regarding the outcome of human pregnancies during which the fetus was exposed to statins is limited and inconclusive (Ofori et al., 2007). In the StAmP trial, pravastatin was chosen because of its highly hydrophilic characteristic and inability to cross the placenta from mother to fetus (Hatanaka, 2000; McTaggart et al., 2001). Several animal studies using the preeclampsia model over-expressing sFlt-1 have now shown that treatment with pravastatin significantly reduces maternal sFlt-1 levels and improves vascular reactivity (Costantine et al., 2010; Fox et al., 2011; Saad et al., 2014). In the RUPP rat model of preeclampsia, treatment with pravastatin also reduced oxidative stress and lowered mean arterial blood pressure in the rats (Bauer et al., 2013). Furthermore, in a rodent model of preeclampsia using a lentiviral vector-mediated placenta-specific expression of sFlt-1 system, treatment of the mice with pravastatin caused a decrease in sFlt-1, but more importantly it significantly raised the level of PlGF in the same mice (Kumasawa et al., 2011). In preeclampsia, the maternal circulating level of PlGF is decreased well before the onset of the disorder (Levine et al., 2004, 2005). The administration of PlGF in lentiviral sFlt-1 infected mice depresses the level of sFlt-1 and ameliorated hypertension, glomerular endotheliosis and proteinuria in the mice (Kumasawa et al., 2011). These observations support the idea that pravastatin increased the level of PlGF and counteracted the effect of sFlt-1, hence improving preeclampsia-like symptoms. The study by Kumasawa and co-workers also proved that treatment with pravastatin did not cause any deformity in the pups (Kumasawa et al., 2011). Although the significance of this decrease in PlGF is unknown it has recently been shown that the loss of hydrogen sulfide (H_2_S) and the H_2_S-producing enzyme cystathionine γ-lyase (Cth) may account for the reduction in PlGF in preeclampsia (Wang et al., 2013). In another study, Fox and colleagues showed that pravastatin increased the eNOS protein expression preferentially in the mice vasculature independent of cholesterol synthesis inhibition. In addition, they reported that pravastatin induced Hmox1 expression in the mice kidney, but not in the liver (Fox et al., 2011). Taken together, these studies support the use of pravastatin as a good candidate for treating or preventing preeclampsia through its ability to restore angiogenic balance to promote vascular health.

### 3.6 Hmox in other pregnancy disorders

Several studies have also attributed the beneficial role of Hmox in other pregnancy disorders. Acevedo and Ahmed showed that Hmox1 and Hmox2 were located in the human uteri and that Hmox is central to the successful outcome of pregnancy through the maintenance of uterine quiescence and the prevention of preterm labor (Acevedo and Ahmed, 1998). Furthermore, the pharmacological inhibition of Hmox1 in pregnant rats resulted in complete fetal resorption (Alexandreanu and Lawson, 2002), whereas adenoviral overexpression of Hmox1 was shown to sustain pregnancy in abortion-prone mice (Zenclussen et al., 2004). In addition, administration of pravastatin rescued placental dysfunction and prevented miscarriages in a spontaneous-abortion mouse model (Redecha et al., 2009).

### 3.7 Clinical perspective

This review has outlined the evidence supporting the notion that activation of the Hmox system by statins could lead to the alleviation of the signs of preeclampsia and consequently prolong affected pregnancies. This would seriously improve outcomes for mothers and babies globally and reduce the lifelong negative health impacts of preeclampsia.

Currently, statins are Food and Drug Administration category X drugs, based on the fact that there are no indications to warrant the use of statins in pregnancy (no benefit to outweigh any risk) and because of a small case series on the teratogenic effects of the original statins in use at the time (Kazmin et al., 2007; Edison and Muenke, 2004). However, a recent observational study on the use of statins in the first trimester of pregnancy looked at 288 women and found no adverse effects (Ofori et al., 2007).

The majority of fetal cholesterol originates from *de novo* synthesis (fetus or placenta) rather than from maternal sources. Fetal mutations in the cholesterol synthesis pathway do not synthesize cholesterol and have very low concentrations of cholesterol in their blood despite normal maternal levels, suggesting a minor role of maternal exogenous sources (Woollett, 2011, 2005). Thus, any concern regarding the use of pravastatin by preeclamptic women, because of its action on maternal cholesterol concentrations, is unfounded. Pravastatin is one of the weakest inhibitors of cholesterol synthesis and it does not cross the placenta, which makes the safety of pravastatin more plausible.

Apart from inhibiting the release of sFlt-1 (Cudmore et al., 2007; Costantine et al., 2010; Fox et al., 2011; Saad et al., 2014), statins have anti-inflammatory and anti-oxidative properties. They increase the activity of thioredoxin (Haendeler et al., 2004), superoxide dismutase (SOD) (Landmesser et al., 2005), and the glutathione peroxidase (Molcanyiova et al., 2006) systems. In addition, statins also improve factors that are compromised in preeclampsia such as NO bioavailability, VEGF, and endothelial progenitor cells (Urbich and Dimmeler, 2005; Cudmore et al., 2007). The world's first randomized placebo-controlled trial, StAmP (Statins to Ameliorate Early Onset Preeclampsia), for the use of statins in early-onset preeclampsia is underway and its outcome will inform obstetricians whether the use of statins in preeclampsia is viable. Women destined to develop early onset preeclampsia could be offered preventive statin therapy or therapies, which up-regulate the “endogenous cytoprotective pathways” like Hmox and Cth. A cheap and widely available therapy against preeclampsia may indeed be on the horizon should the StAmP trial prove successful and if a worldwide randomized controlled trial for the use of pravastatin to treat preeclampsia is positive. This could reduce worldwide maternal and infant mortality associated with preeclampsia and would significantly move forward the United Nations Millennium Development Goal (MDG-4, reducing maternal mortality, and MDG-5, reducing child mortality) set in 2000 following the Millennium Summit of the United Nations.

In order to develop other therapies apart from statins to tackle preeclampsia, basic research is still needed to elucidate fully the role of Hmox1 and its metabolites. Murine models do not equate to preeclampsia in women, but they can replicate many of the preeclampsia symptoms and thus offer very useful tools for performing proof of principle experiments to determine the role of specific genes and therapies. What is now needed is the use of sophisticated knockout and tissue-specific knockdown mouse models combined with further clinically based studies, as has been the case in the cardiovascular field.
